# Census-Dependent Mortality of Ventilated Patients With COVID-19 in Israel: Noninterventional Observational Cohort Study

**DOI:** 10.2196/41749

**Published:** 2024-07-09

**Authors:** Joseph Mendlovic, Francis B Mimouni

**Affiliations:** 1 Shaare Zedek Medical Center Affiliated with the Hadassah-Hebrew University School of Medicine Jerusalem Israel; 2 Associate Director General Office Israel Ministry of Health Jerusalem Israel; 3 Faculty of Medicine Tel Aviv University Tel Aviv Israel; 4 Leumit Health Service Tel Aviv Israel

**Keywords:** COVID-19, mortality, ventilation, intensive care, pandemic, contagious, disease, mortality, database, data, patient, mortality, medical, resources, validation, public policy, policy, pandemic, health policy, global health policy

## Abstract

**Background:**

The COVID-19 pandemic led to several surges in the mass hospitalization rate. Extreme increases in hospital admissions without adequate medical resources may increase mortality. No study has addressed the impact of daily census of ventilated patients on mortality in the context of the pandemic in a nationwide setting.

**Objective:**

This study aimed to determine whether daily census of ventilated patients affected COVID-19 mortality rates nationwide in Israel.

**Methods:**

We conducted a cohort study using nationwide, public-domain, population-based COVID-19 data of hospitalized patients from an Israeli database from March 11, 2020, until February 11, 2021. We included all COVID-19 hospital admissions, classified as mild to severe per the Centers for Diseases Control and Prevention classification irrespective of whether they were mechanically ventilated. Outcome measures were daily death rates and death rates expressed as a percentage of ventilated patients.

**Results:**

During the study period (338 days from March 11, 2020, to February 11, 2021), 715,743 patients contracted and were clinically confirmed as having COVID-19. Among them, 5577 (0.78%) patients died. In total, 3398 patients were ventilated because of severe COVID-19. Daily mortality correlated with daily census of ventilated patients (*R*^2^=0.828, *P*<.001). The daily percent mortality of ventilated patients also correlated with the daily census of ventilated patients (*R*^2^=0.365, *P*<.001)—backward multiple regression analysis demonstrated that this positive correlation was still highly significant even when correcting for the average age or gender of ventilated patients (*R*^2^=0.4328, *P*<.001) or for the surge in their number. Overall, 40% of the variation in mortality was explained by variations in the daily census of ventilated patients. ANOVA revealed that at less than 50 ventilated patients per day, the daily mortality of ventilated patients was slightly above 5%, and it nearly doubled (10%) with 50-149 patients; moreover, in all categories of ≥200 patients ventilated per day, it more than tripled at ≥15% (*P*<.001).

**Conclusions:**

Daily mortality rates per ventilated patient increased with an increase in the number of ventilated patients, suggesting the saturation of medical resources. Policy makers should be aware that expanding medical services without adequate resources may increase mortality. Governments should perform similar analyses to provide indicators of system saturation, although further validation of these results might be needed to use this indicator to drive public policy.

## Introduction

SARS-CoV-2, the causative agent of COVID-19, was first identified on November 17, 2019, and was declared a pandemic by the World Health Organization on March 11, 2020 [1]. The virus is extremely contagious and the emergence of multiple mutated strains increased its contagiousness [2]. The disease’s severity, as observed in a small percentage of patients, warrants complex intensive care facilities [2]. At the onset of the pandemic, medical systems worldwide have been tremendously challenged by COVID-19, leading to major disruptions in routine hospital services, leading to chaos and exhausting reserve medical supplies [3-6]. Excess mortality beyond expected rates has been observed in many countries, including Israel [7]. Possibly, many potentially curable patients might have died because medical services were overwhelmed [6-8]. However, the extent of the impact of the saturation of medical services on a country’s COVID-19 mortality has not been systematically studied, and no study has addressed the impact of census on mortality in the context of the pandemics in a nationwide setting. The existence of such a relationship may be suggested by the strong correlation reported between the number of hospital beds per population size and COVID-19–specific mortality both in the United States [9] and worldwide [10]. In Israel, the number of intensive care units (ICU) beds is much lower than that in other high-income countries such as Germany or the United States [11,12].

We analyzed the national database of the Israeli Ministry of Health (MOH), systematically collected and reported since March 11, 2020, until February 11, 2021, that is, prior to mass vaccination conducted in Israel.

We hypothesize that daily COVID-19 mortality rates would be directly related to the daily national census of ventilated patients, increasing each time with an increase in the number of ventilated patients. Alternatively, it was also possible that because of routine experience in treating ventilated patients in regular wards, there would be no discernable effect of workload on mortality.

### Methods

#### Study Design

In this retrospective cohort study, data were extracted from a nationwide, official, open-access COVID-19 database of Israel [13] and the MOH [14], specifically constructed for the purpose of reporting and research.

#### Setting

We used a national, public repository database, which was curated and made available on a government website to the general public for free use.

We collected data from all 31 general public hospitals in Israel, whereby clinical data were reported to the MOH 3 times daily. Data were uploaded automatically through a dedicated interface. After processing and quality control testing, it became possible to obtain a nationwide perspective of hospitalized patients. Simultaneously, with automatic transfer of information, manual reports were also transmitted from hospitals for the purpose of quality control, as described below. Data for a particular data were finalized, and updated data were uploaded into the system by midnight of any particular day. These databases were updated every weekday, 3 times daily. The data included the daily cumulative number of patients having tested positive for COVID-19, new laboratory-proven cases, vaccinated people (newly and cumulative), hospitalized patients (with mild, moderate, or severe COVID-19), mechanically ventilated patients with their average age and gender, and COVID-19–related deaths.

In addition to hospital-generated data, laboratory data of all new patients were regularly uploaded to the database through specific interfaces between testing laboratories and MOH computers. In Israel, SARS-CoV-2 positivity was confirmed via polymerase chain reaction–based swab tests using samples obtained from both the throat and one nostril in each patient. Testing was conducted at designated testing sites, such as task-specific health maintenance organizations’ clinics converted to testing sites, task-specific testing tents, mobile vans, and Home Front Command run testing compounds, in association with Magen David Adom (the Israeli “Red Cross”) and health maintenance organizations. In addition, patients in isolation or those who were unable to reach the testing site for medical reasons were tested at home. These laboratories reached a peak of >120,000 tests per day, while the Israeli population is approximately 9,300,000 [13].

To fully understand the settings of this study, one must be aware of how the pandemic was handled in Israel. The Israeli national health system is highly centralized and is under strict MOH regulation. During the pandemic, and in view of the chaos publicized in many countries whose medical systems were overwhelmed, the MOH took the following measures (among others): (1) throughout the whole pandemic, it facilitated the redirection of critically ill patients among medical centers to distribute the patient burden evenly; (2) all elective surgeries were canceled to focus hospital activities on patients with COVID-19—the latter requirement was strictly followed during the first peak of the pandemic, and was thereafter canceled to prevent potential harm; and (3) during surges in the number of hospitalized patients, care of critical patients (both ventilated and nonventilated) was provided by additional physicians and nurses who are not related to anesthesiology or ICU departments but have previous experience of handling ventilated patients in non-ICU wards; indeed, there are not enough ICU beds in Israel to take care of all ventilated patients, and many of them are cared for in non-ICU wards [15]. Owing to the scarcity of experienced physicians and nurses (some of whom were in quarantine due to unprotected, inadvertent exposure to SARS-CoV-2) during peaks of the COVID-19 pandemic, additional medical personnel were assigned to coronavirus wards after receiving a “crash” course on ventilator management. In Israel, ICU beds are usually occupied at a rate of approximately 100%, and the overflow is taken care of on regular wards. During the pandemic, most critically ill patients with COVID-19 ended up being cared for in satellite units (designated coronavirus wards) because of 100% occupancy rates of formal ICUs, and to facilitate the isolation of these patients. Importantly, in Israel, there are no hospitals such as the so-called “community hospitals” (level 1 hospitals) present in the United States, which do not contain ICUs; thus, all hospitals contributing to this database are regional, tertiary care centers.

The period of data collection in this study was from March 11, 2020, when the database was implemented, until February 11, 2021. All hospitalized patients were followed up from exposure and until death or discharge from the hospital (hereinafter the “follow-up period”). As part of quality control processes, a comparison was made between automatic and manual reports. Gaps were checked manually and corrected as needed. Additional checks were carried out for deceased patients, and hospital reports and death certificates were additionally compared to verify that the main cause of death was COVID-19–related.

#### Participants

The participants in this study were all the patients who tested positive for COVID-19 at some point during the study period. They were selected in accordance with a laboratory-confirmed diagnosis as described above. The patients were classified by the severity of symptoms: mild, moderate, and severe. Initially, each hospital report used its own criteria for levels of severity. From July 12, 2020 (day 123 of the study period since the implementation of the database on March 11, 2020), the MOH issued specific definitions established by the National Institutes of Health [16,17]: ventilated patients were those requiring invasive mechanical ventilation (with an endotracheal or tracheostomy tube). Mild disease was defined as laboratory confirmation in combination with mild symptoms (fever, cough, weakness, and loss of taste and smell). Moderate disease was defined as laboratory confirmation together with pneumonia (clinical or through imaging). Severe disease was defined as laboratory confirmation with one or more of the following: a respiratory rate of >30 breaths per minute, oxygen requirement of >30%; oxygen saturation in arterial blood of ≤93% in ambient air, and a ratio of arterial oxygen pressure and oxygen requirement of <300.

#### Follow-Up Methods

The number of deaths was updated daily. The overwhelming majority of patients who died were invasively ventilated prior to dying, and several of them were even placed on mechanical ventilation during transport to the hospital. We cannot, however, rule out that some patients died prior to arriving at the hospital; nonetheless, they were customarily ventilated by paramedics en route to the hospital unless they belong to the very small group of “do not resuscitate” patients because they have an incurable disease that would potentially lead to their death within less than 6 months (according to the Israeli “Dying Patient Law”).

#### Assessments

The main outcomes of interest were whether the patient died or not, was ventilated or not, their length of stay (LOS) in the hospital, and the duration for which they received mechanical ventilation. Potential confounders, predictors, and effect modifiers were the only variables that were prospectively collected from this database, namely gender, patients’ age, and the daily census of ventilated patients in Israel.

#### Data Source

The data source is the Israeli MOH’s National COVID-19 Database, systematically collected and reported since March 11, 2020, until February 11, 2021, that is, prior to mass vaccination that occurred in Israel.

#### Ethical Considerations

The website we used is a public repository, available free of charge to the public. It is completely anonymized and deidentified; therefore, it was appropriate to not apply for approval from an ethics review board. Under the federal regulations for human subjects research (45 CFR Part 46), research involving publicly available data sets would not require review by an institutional review board—no application is required—as long as the data are obtained from sources that are publicly available and are deidentified and uncoded as in this study [18]. Nevertheless, since the data were collected in Israel, the National Committee for Human Medical Research of the Israeli MOH provided its full approval for the study and waived the requirement for obtaining informed consent. The Israeli National Committee for Human Medical Research deemed this study exempt from ethical approval since this study involves public data processing for the purposes of policy making and reflecting on the national system’s dealings with the epidemic; therefore, it does not require to adhere to the tenets of the declaration of Helsinki.

#### Statistical Analysis

The Minitab Statistical Package (version 16; Minitab, LLC) was used for analyses. Data were tested for normality and expressed as mean (SD) or median (IQR) values as requested. Stepwise backward multiple regression analysis was carried out to determine the correlation between daily percent mortality per group of ventilated patients (dependent variable) and the daily census of ventilated patients (independent variable), while taking into account potential confounding variables that may affect daily percent mortality, such as mean patient age, gender, and COVID-19 surge (surges 1, 2, or 3). This method places all the independent variables at once in the equation, and eliminates those found to be insignificant sequentially step by step, repeating the operation each time until only the significant variables remain in the final equation. This method allows for results that are not influenced by the order of introduction of the independent variables. Daily percent mortality per group of ventilated patients was calculated as the daily mortality rate in Israel divided by the census of ventilated patients on the same day. Independent variables entered in the regression equation were only those found to influence (in univariate analysis) the daily percent mortality of ventilated patients at an α value of <.10.

Analysis of means using ANOVA was carried out to determine differences in the mortality rates of ventilated patients by group based on their number in the daily census. We arbitrarily analyzed daily census according to 8 groups of increasing census size: group 1 containing 1-49 ventilated patients, group 2 containing 50-99 ventilated patients, group 3 containing 100-149 ventilated patients, group 4 containing 150-199 ventilated patients, group 5 containing 200-249 ventilated patients, group 6 containing 250-299 ventilated patients, group 7 containing 300-349 ventilated patients, and group 8 containing 350-399 ventilated patients.

No sample size calculation was carried out as we used all the available numbers nationwide.

## Results

During the study period (338 days from March 11, 2020, to February 11, 2021) 715,743 patients contracted and had a laboratory-confirmed diagnosis of COVID-19. They constitute the study population. Among them, 5577 (0.78%) patients died. In total, 3398 patients were ventilated because of severe COVID-19. We retrieved complete data (including outcomes, gender, age, and LOS in hospital) for 3373 of them (as of this writing, there was no determined outcome for 25 patients who are still ventilated). These data are presented in Table 1. Briefly, patients who died were older, and there were many more men ventilated than women, but mortality among ventilated men and women was similar (approximately one-third of patients). LOS in hospital was much shorter among patients who died than among those who survived. The LOS to ventilation (from hospital admission to the end of hospital stay) was shorter for patients who died, and the LOS from ventilation to outcome (discharge or death) was much longer for survivors.

**Table 1:** Demographic data for ventilated patients (N=3373).

	Age^a^ (years), mean (SD; median; IQR)	Gender^b^ (male:female), n:n (% males)	Length of stay to outcome (days)^a^, mean (SD; median; IQR)	Length of stay to ventilation (days)^a^, mean (SD; median; IQR)	Length of stay from ventilation to outcome (days)^a^, mean (SD; median; IQR)
Alive (n=1128; 33.44%)	60.3 (16.3; 62; 51-71)	721:407 (63.9%)	35.7 (29.4; 27; 15-47)	4.9 (5.9; 3; 1.6)	30.8 (28.4; 22; 11-42)
Dead (n=2245; 66.56%)	72.2 (13.0; 74; 65-82)	1487:758 (66.2%)	18.7 (16.5; 15; 8-25)	6.0 (6.6; 4; 1-8)	12.8 (15.1; 8; 3-17)

^a^*P*<.001.

^b^*P*=.18.

**Figure 1:**
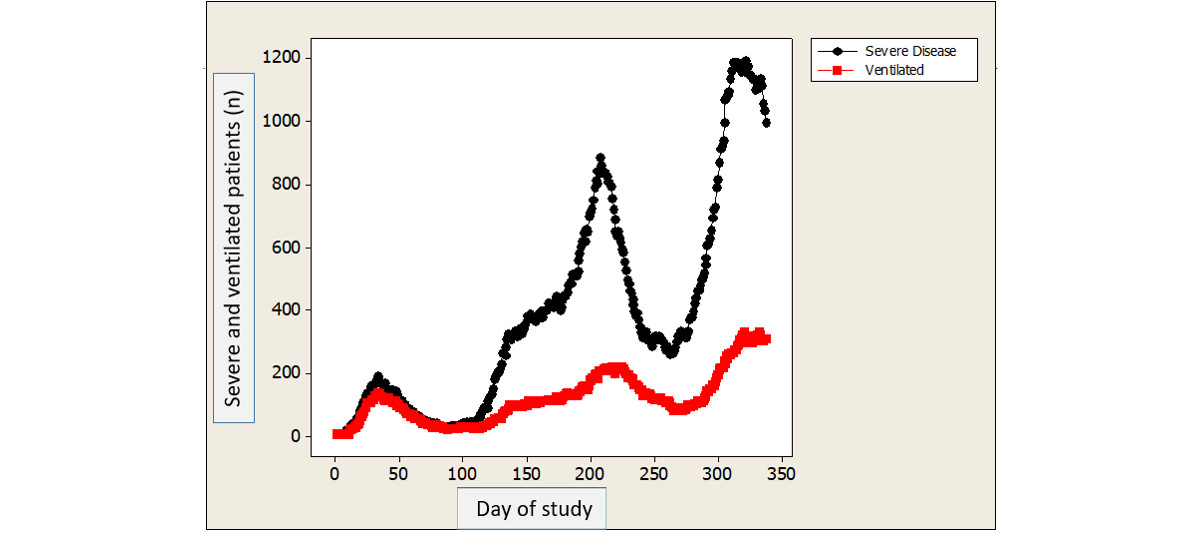
Daily rates of patients with severe COVID-19 (black dots) and those ventilated (red dots) over time.

**Figure 2:**
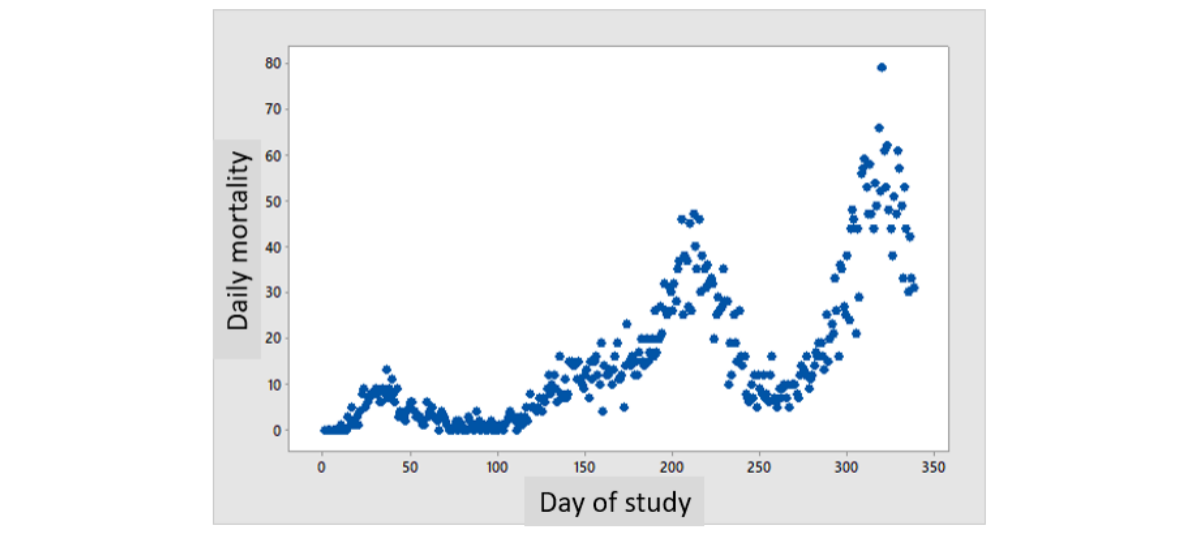
Daily mortality over time.

**Figure 3:**
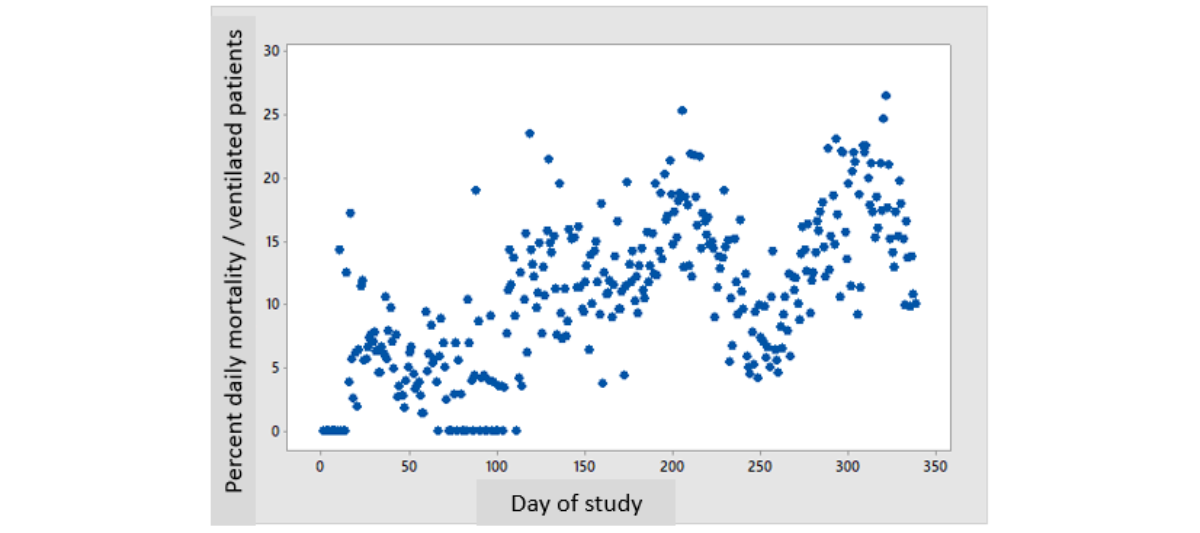
Daily percent mortality of ventilated patients over time.

Figure 1 depicts the daily number of patients hospitalized every day of the study for severe disease and the number of ventilated patients, showing 3 surges of frequencies. Figure 2 depicts the daily mortality per day of the study, which also shows 3 surges of daily death rates, parallel to the surges in the number of patients with severe disease and ventilated patients. Figure 3 shows the daily percent mortality of ventilated patients over time, which also follows a similar pattern to the 3 peaks.

**Figure 4:**
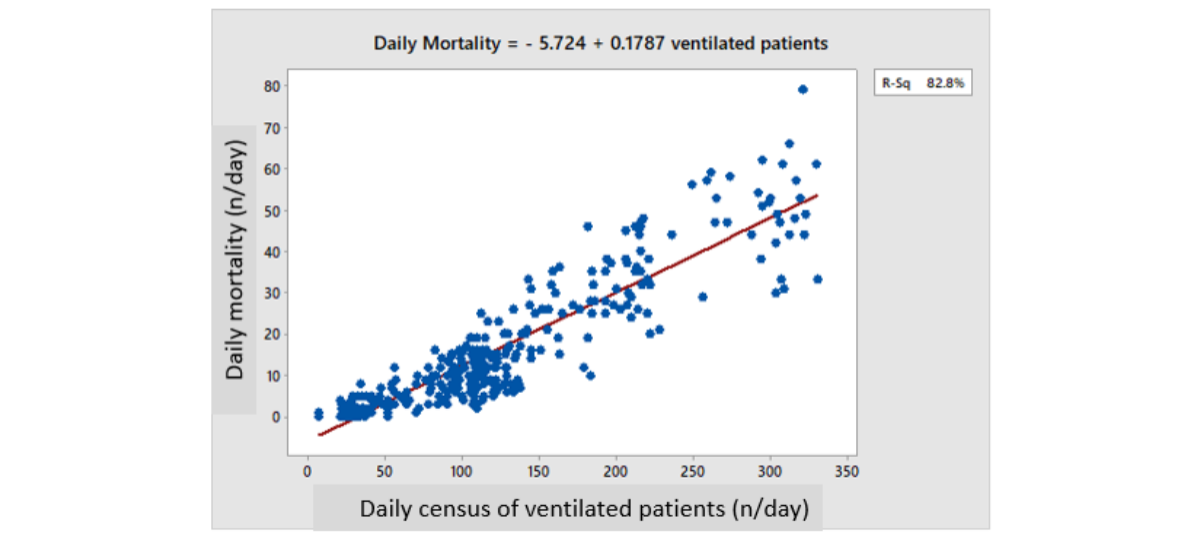
Daily mortality rates versus the daily census of ventilated patients.

**Figure 5:**
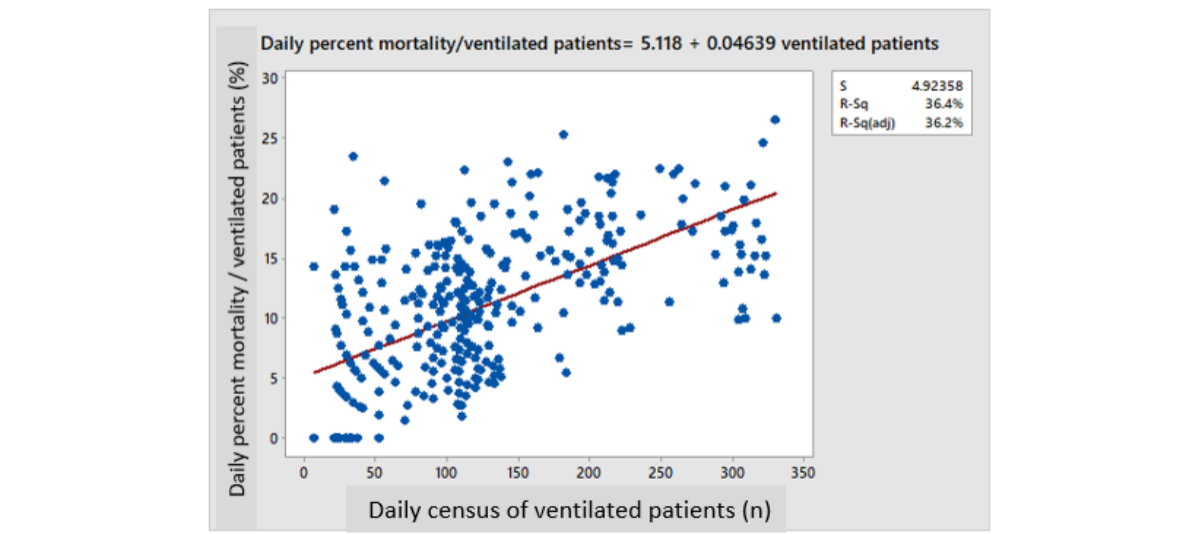
Daily percent mortality of ventilated patients versus daily census of ventilated patients.

Daily mortality correlated with the daily census of ventilated patients (*R*^2^=0.828, *P*<.001; Figure 4). The daily percent mortality of ventilated patients also correlated with the daily census of ventilated patients (*R*^2^=0.365, *P*<.001; Figure 5). Backward multiple regression analysis revealed that the latter positive correlation was still highly significant even when correcting for the average age or gender of ventilated patients (*R*^2^=0.4328, *P*<.001) or for the wave number (*P*>.05). Both average age and gender remained significant (*P*<.001) in the final analysis (older age and a higher percentage of females contributing to higher mortality; Table 2). The *R*^2^ value of the correlation equation predicting the percentage mortality per ventilated patient was at best 0.4, implying that 40% of the variation in mortality can be explained by variations in the daily census of ventilated patients. Obviously, the rest or the variability (60%) was explained by other factors that were probably patient-dependent (such as BMI, diabetes, or other chronic diseases), which were not retrievable from this database.

**Table 2:** Multiple regression analysis results showing the relative contribution of the daily census of ventilated patients, gender, and age (independent variables) on the daily percent mortality of ventilated patients (dependent variable).

	Daily census of ventilated patients^a^	Gender^a^	Age^a^	Model summary
Partial *R*^2^	0.3574	0.0275	0.0005	0.3915
Effect size (adjusted sum of squares)	3772.7	290.7	68.6	5012.5
*P* value	<.001	<.001	.09	<.001

^a^All variables here refer to the dependent variable, that is, daily percent mortality of ventilated patients.

**Figure 6:**
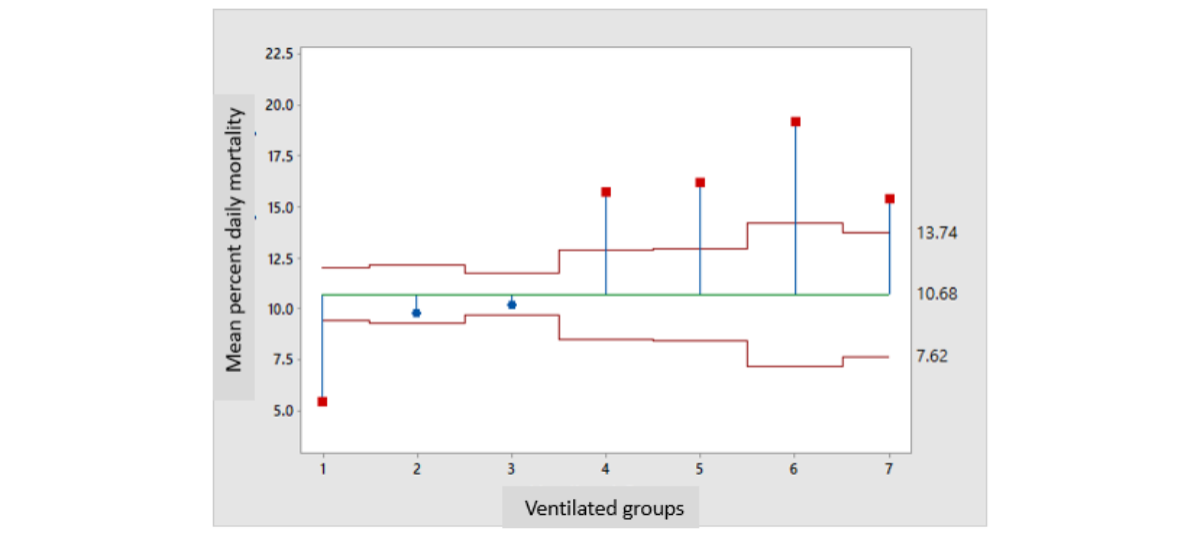
Analysis of the mean percent mortality of ventilated patients by ventilated patients' groups (group 1: 1-49; group 2: 50-99; group 3: 100-149; group 4: 150-199; group 5: 200-249; group 5: 250-299; group 6: 300-349; group 7: 350-399).

ANOVA (Figure 6) revealed that with <50 ventilated patients per day, the daily mortality of ventilated patients was slightly above 5%; with 50-149 patients, it nearly doubled (10%); and in all 3 categories of 200 and more patients, it more than tripled at ≥15%.

We investigated whether an increase in the number of ventilated patients during a specific surge may have been related to an accumulation of mechanically ventilated patients from previous surges. In fact, LOS or length of ventilation did not differ among patients who were admitted during surges 1, 2, or 3 (ANOVA *P*>.05). Moreover, among 11 of 164 patients admitted to hospital during surge 1 died during surge 2, and none died during surge 3; 65 of the 1088 patients admitted to hospital during surge 2 died during surge 3 (together with 993 patients admitted to hospital during surge 3)—among them, the vast majority (n=63) were admitted within the last 20 days of surge 2.

## Discussion

### Principal Findings

This is the first nationwide study demonstrating increasing mortality with increasing demand for health care resources during the COVID-19 pandemic. Indeed, daily mortality rates expressed as a percentage of ventilated patients correlated with the daily census of ventilated patients. We do not believe that this increase in percent mortality rates during surges was due to the accumulation of mechanically ventilated patients who stayed alive until but died during the following surge because the LOS or length of ventilation until death did not differ among patients who were admitted during surges 1, 2, or 3, and also because such “leftovers” were much less than 10% of a given surge.

In China, higher mortality was recorded in Wuhan than in other Chinese provinces, which is related to the rapid escalation in the number of infected patients with insufficient access to health care resources [19]. In the United States, a retrospective cohort study of a 26-hospital–integrated delivery system showed an association between a greater percentage of COVID-19–related admissions of hospital capacity and a lower survival rate [20]. There are multiple examples in the news media describing “accidental deaths” due to improper care of patients with COVID-19 [21]. To limit the dimensions of this catastrophe, many countries had imposed lockdowns and quarantines, leading to consecutive peaks of the pandemic [21].

Israel is generally considered to be technologically sophisticated [22], but its medical system is greatly stretched [23]. In a 2020 report from the 37 nations belonging to the OECD (Organisation for Economic Co-operation and Development), Israel features among the bottom 4 nations in terms of acute hospital care beds (2.2 per 1000 population), which is well below countries such as Japan (7.8 per 1000 population) [23]. Israel also features among the 11 countries with the lowest numbers of both physicians and nurses per 1000 population [23]. The Israeli system considers itself efficient, reporting daily occupancy rates among the highest worldwide, at 93.3% on average, second only to Ireland among the 37 OECD countries [23]. In Israel, the number of ICU beds is 4.6 per 100,000 population and 2.2 ICU beds per 100 hospital beds (reported in 2007) [11], which is much lower than that in other high-income countries such as Germany (24.6 hospital beds per 100,000 population and 4.1 ICU beds per 100 hospital beds), or the United States (20 hospital beds /100,000 and 9 ICU beds per 100 hospital beds) [12]. In Israel, every winter, many patients are ventilated in internal medicine wards because of the unavailability of ICU beds [15]. However, in non–COVID-19 times, the mortality of ventilated patients in non-ICU wards was higher than that of formal ICU beds, proving that the system might be “cheaper” but not necessarily better [15]. There is a strong correlation between the number of hospital beds per population size and COVID-19–specific mortality both in the United States [9] and globally [10]. This does not imply that *more* is necessarily *better*. Indeed, case-mix and ICU organization are important to consider. Strategies for how to use ICU beds, the proportions of mechanically ventilated versus nonventilated patients, the availability of intermediary care, and other factors are important to consider. For instance, if a country has a policy of admitting nonventilated patients to high- or medium-care departments instead of ICUs or transferring extubated patients to intermediary care units immediately, such a country will need fewer ICU beds than those that do the opposite.

Daily mortality expressed as a percentage of ventilated patients correlated with the daily census of ventilated patients. This was true even after taking into account the average age of ventilated patients and their gender or surge number. We suggest that the medical system reached saturation, that is, its inability to adequately handle complex ventilated patients. The reasons for this saturation are multiple. We cannot currently determine whether or not there was a shortage of specific drugs, mechanical ventilators, oxygen, or other supplies, or a shortage of personnel, higher patient-nurse ratios, lack of ICU beds, etc, but these have been reported anecdotally in the general nonmedical literature [24]. In fact, it is highly possible that whenever the census of ventilated patients increased, some patients died because they could not receive proper care and not because their disease was incurable. These data should be considered within the context of the quasi-heroic behavior of exhausted, overworked teams of caretakers that performed their duties throughout the pandemic under perilous circumstances. Furthermore, it is highly possible that parallel increases in the mortality of mechanically ventilated patients without COVID-19 occurred during the same periods. Unfortunately, no similar database for was maintained for patients without COVID-19, and we were not able to verify if this occurred.

In this study, mortality was observed among approximately two-third of patients—a number difficult to compare to that of other countries since reported death rates of ventilated patients with COVID-19 is also dependent upon case-mix, varying from 48% among younger patients (younger than 40 years) to 84% among older ones (>80 years of age) [25]. It has recently been shown that for instance, the physical manifestations of frailty and comorbidity, particularly a history of cognitive impairment and falls, may be useful in identifying patients with COVID-19 who need additional support during hospitalization and may be at a higher mortality risk [26]. This may also be dependent on noninvasive and invasive ventilation strategies. A recent meta-analysis by Lim et al [26] estimated the mortality rate among such patients to reach 45% on average, which is lower than that reported in this study. In our study, the effect of the census of ventilated patients census on daily mortality was nearly identical during the 3 different surges, which suggests that the SARS-CoV-2 variants, likely to differ among the various waves, affected mortality in a similar manner.

Worldwide, there have been reports of “spontaneous” reductions in critical care admissions, such as those related to stroke and cerebral emergencies [27], accidents, emergency surgery, and acute coronary events [28-30], which may have reduced the ICU burden. We suspect that it may have existed in Israel as well in particular during the first surge, but it did not reduce the ICU burden to a point that prevented an increase in mortality rates reported here. A recent study reported that the management of critically ill patients with COVID-19 in the United Kingdom was far from ideal in numerous cases, with systematic errors in the measurement of height and derived ideal body weight and delayed applications or nonimplementation of evidence-based interventions for acute respiratory distress syndrome (in particular, prone positioning) [31].

Our findings may not be exclusive to the COVID-19 pandemic. For instance, Israel has not yet achieved peace with all its neighbors, and a large-scale increase in hostilities may have led to a number of casualties that the Israeli medical system might not be able to handle. Israel is also located on The Great Syria-African Rift and is at risk of major and potentially deadly earthquakes to occur.

The major strengths of this study are the use of a national large database, a long study period for most part of the pandemic, and the measures undertaken for quality control. A limitation of this study relates to changes in some definitions in the middle of data collection. These changes are unlikely to influence our main findings, in that mortality rates (regardless of severity staging) correlated with the workload of ventilating patients. Another limitation of this study is that the data set did not evaluate workload in individual hospitals and individual ICUs (occupancy rates, staffing patterns, and hours of nursing care per patient per day) relative to resource availability, which somewhat limits drawing causal inferences. It is unclear whether our findings are universal or change when examined in accordance with a hospital’s geographic location or size; however, of note, during each surge, the Israeli MOH frequently intervened and helped individual hospitals to move patients between hospitals to prevent maldistribution. We also provide no data on ventilated patients in non–COVID-19 beds, whose survival might also have been affected by the shift of many medical resources from other departments to care for patients with COVID-19; hence, we were unable to provide data on noninvasive ventilation or the use of vasoactive drugs or even extracorporeal membrane oxygenation that was administered to some patients. This information would have potentially helped stratify the complexity of health care requirements but was not available in this database. Finally, outcomes noted were either discharge from hospital or death, while some patients may have died after discharge. We do not believe that withholding strategies due to lack of bed availability may have influenced survival since in Israel, since patients requiring ventilation are not dependent upon bed availability, and are ventilated in non-ICU settings. Additionally, the law in Israel does not allow extubating of a dying patient.

### Recommendations to the Health Care Leadership

Recognizing the fact that the ability to provide adequate intensive care and respiratory care services is a critical and unique national resource during pandemics and other emergencies. The use of percentage mortality or the census of ventilated patients as a potential key tool for monitoring the hospital system nationwide.

An information system should be constructed such that it supports the provision of a nationwide perspective on all ICU beds in Israel so as to divert patients in emergencies where there is an unusual load while simultaneously taking advantage of the relatively short geographical distances in the country.

A multiyear program for intensive care training for medical and nursing teams should be developed, while finding solutions that enable maintaining an adequate professional level even for teams that have undergone intensive care training but do not regularly work in ICUs.

### Conclusions

Since this study is a noninterventional observational study, the correlations we found (ie, an increase in the percent mortality of ventilated patients with an increase in the census of ventilated patients) are concerning, and suggest, but do not prove, causality. We speculate that the results of this study might help health policy makers to address medical capacities on a nationwide scale, determine how much their system has been affected by pandemics, and to what extent it should be strengthened by the addition or expansion of intensive care facilities. This should facilitate better preparation for future pandemics, the appearance of the next mutant of SARS-CoV-2 that may be resistant to currently available vaccines, or future events potentially resulting in a burden on the health care system. Obviously, there should be a balance between the needs of a country under extraordinary circumstances and needs during “routine” circumstances. An increase in the number of beds might not be the only way out, especially when there are no trained personnel to take care of patients. Increasing ICU training and ICU rotations of health care workers might better prepare a country to handle a large-scale catastrophe.

## Abbreviations

ICU: intensive care unit
LOS: length of stay
MOH: Ministry of Health
OECD: Organisation for Economic Co-operation and Development

## Data Availability

Data are available on the Ministry of Health's World of Data’s website [32].
